# Suicide among people treated for drug use disorders: a Danish national record-linkage study

**DOI:** 10.1186/s12889-020-8261-4

**Published:** 2020-01-31

**Authors:** Morten Hesse, Birgitte Thylstrup, Abdu Kedir Seid, Jens Christoffer Skogen

**Affiliations:** 1 Center for Alcohol and Drug Research, Aarhus University, Aarhus, Denmark; 2 Department of Health Promotion, Norwegian Institute of Public Health, Bergen, Norway; 3 Alcohol & Drug Research Western Norway, Stavanger University Hospital, Stavanger, Norway; 4 Department of Public Health, Faculty of Health Sciences, University of Stavanger, Stavanger, Norway

**Keywords:** Drug use disorder, Suicidal behaviour, Mental health, Treatment, Longitudinal study, Cohort, Competing risks

## Abstract

**Background:**

Substance use disorders are a major risk factor for suicide. However, less is known about specific risk factors for suicide in people with substance use disorders.

**Methods:**

This population cohort study assessed suicide among people treated for drug use disorders in Denmark 2000–2010, and described risk factors for completed suicide. Data from 27,942 individuals enrolled in treatment were linked to national registers and matched with controls without drug use disorder and with (*n* = 138,136) or without psychiatric history (*n* = 1574). Competing risk regression was used to identify risk factors of completed suicide.

**Results:**

There were 163 suicides among patients with a history of drug treatment (0.6% of patients). Increased risk was associated with younger age at enrolment (hazard ratio [HR] = 0.97, 95% confidence interval (CI): 0.95, 0.98), history of psychiatric care (HR = 1.96, CI 95%: 1.39, 2.77), opioid use (HR = 1.81, 95% CI: 1.23, 2.68), and alcohol use (HR = 1.56, 95% CI: 1.09, 2.23). Lower risk was associated with cannabis use (HR = 0.69, 95% CI: 0.50, 0.96). Compared with age- and gender-matched controls without a history of treatment for substance use disorders or recent psychiatric care, the standardized mortality ratio due to suicide was 7.13 for people with drug use disorder without a history of psychiatric care (95% CI: 5.81, 8.44), 13.48 for people with drug use disorder and psychiatric history (95% CI: 9.75, 17.22), and 13.61 for people with psychiatric history only (95% CI: 6.72, 20.50).

**Conclusions:**

Risk of suicide is increased among people with drug use disorders. Access to treatment for co-morbid mental health problems for people with drug use disorders could potentially reduce risk of suicide.

## Background

Suicide is a global phenomenon; nearly 800,000 people die from suicide every year, with a higher prevalence among men compared to women, peaking between the age of 15 and 24 among women, and between the age of 25 and 45 among men [1]. According to the 2016 Global Burden of Disease Study, suicide was among the top ten leading causes of death in Western European countries [2].

Several meta-analytic studies have reported an association between substance use, including alcohol and illicit drug use on the one hand, and suicidal ideation, suicide attempts, and suicide death on the other [3, 4]. A recent meta-analysis found that in case-control psychological autopsy studies, the presence of a drug use disorder (DUD) was associated with a 7-fold increase in suicide [5]. Further, previous studies have reported significant associations between the misuse of prescription drugs and suicidal ideation or suicide [6, 7].

### Risk factors related to suicide

Significant risk factors for suicidal behavior include misuse of alcohol or drugs, a history of self-harm and attempted suicide, and psychiatric disorders [8–13]. Globally, alcohol dependence (13.3%), amphetamine dependence (2.4%), opioid dependence (1.9%) and cocaine dependence (0.9%) are important attributable factors for suicide [14]. In Denmark, alcohol use was attributable to 33.3% of deaths due to suicide in 2017, while 3.1% were attributable to drug use according to global burden of disease estimates [15]. Cannabis use and cannabis use disorders have been shown to be correlated with suicidal behaviour and ideation, although there is no convincing evidence that the link is causal [16]. It is likely that the strong association between cannabis and suicide could be explained by other factors, such as co-morbid mental health and behavioural problems [17].

Previous research shows that mood and anxiety disorders are risk factors of suicide [18], including both unipolar and bipolar mood disorders [19], especially in the early phases of illness [20]. In addition, borderline personality disorder is associated with an elevated risk of completed suicide, especially among individuals with multiple hospitalizations [21].

Despite the large body of research on suicide and substance use and psychiatric co-morbidity, relatively few studies have assessed predictors of suicide in people with psychoactive DUD. A recent study found that both self-reported psychiatric symptoms and psychiatric treatment history independently predicted completed suicide among people with DUD [22]. However, beyond this single study, there is a paucity of research that has examined risk factors associated with suicide completion in individuals with DUD.

The present study had two aims: [1] to assess excess mortality due to suicide among people treated for DUD in Denmark, and [2] to describe socio-demographic and clinical risk factors associated with completed suicide.

## Methods

### Data

The present study used data from multiple Danish national registers, all of which are continuously updated.

*The Civil Registration System* was established in 1968 and includes unique individual identification number, name, gender, date of birth, place of birth and residence, citizenship, identity of parents and spouses [23].

*The Registry of Drug Abusers in Treatment* has been recording information on people seeking treatment for DUD in publically funded treatment centers under the Danish social services since 1996 [24].

*The National Patient Register* was established in 1977 and contains personal and admission data for secondary care. The personal data include the unique identification number, municipality, and region of residence. The admission data include hospital and department codes, admission type, patient contact type (inpatient, outpatient, or emergency department), referral information, contact reason, and dates of admission and discharge [25].

*The Psychiatric Central Research Register* has recorded episodes of psychiatric care since 1970, and contains information for all outpatient, inpatient, and emergency contacts at psychiatric hospitals, including dates of beginning and end of treatment, diagnoses, type of referral, place of treatment, place of residence, and mode of admission [26].

*The Danish Registry for Causes of Death* contains information on dates and causes of death based up on the death certificate. Since 1875, the Danish National Board of Health has maintained the registers covering deaths among all Danish residents dying in Denmark, and since 1970 such records have been computerized [27].

*The Central Criminal Register* contains information about offenses and offenders in criminal cases for use in criminal procedures since November 1978. Information is transferred from the central crime register to Statistics Denmark [28].

All registers were linked, using the unique identification number assigned to each individual up on birth or first entry to Denmark as an immigrant.

The data for this study are stored on secure servers at Statistics Denmark, and all procedures were approved by the Danish Data Protection Agency. Since the data used for this study were collected and stored for monitoring and quality assurance, no ethics evaluation was needed under Danish law.

### Inclusion criteria

Patients were included in the study if they had been enrolled in a publicly funded outpatient treatment facility for DUD in Denmark between 2000 and 2010, and were in the age range of 18 to 75 years at time of admission. Patients were excluded if their date of death was invalid. Less than five cases were omitted from the analyses, as their date of death was recorded as January 1st 1960, although they had been in treatment after January 1st 2000 (the exact number cannot be given due to data protection rules prohibiting the download of microdata). We followed the patients over the entire observation period, beginning from first registered treatment enrolment to completed suicide or December 31, 2010, whichever occurred first.

### Measures

#### Outcome variable

The outcome in the study was defined as time from the first registered enrollment at a treatment center for DUD to completed suicide. Dates and causes of death were identified using the Danish Register of Cause of Death. We used ICD-10 codes to identify all completed suicides ascribed to intentional self-harm (X60-X84) or the sequelae of intentional self-harm (Y87.0) [29].

#### Predictors

The information on all substances used by the patients in the twelve months prior to enrolment in treatment were extracted from The Registry of Drug Abusers Undergoing Treatment. These variables were dummy-coded for the following types of substances: any opioids; central stimulants; cannabis; any recorded problem drinking in the database; use of benzodiazepines; methyl​enedioxy​methamphetamine; and intravenous drug use. Further, we used a categorical predictor indicating previous drug treatment versus no previous drug treatment, or missing information on previous treatment based on the admission form. Using the code of reason for contact from the National Patient Register, we constructed a dummy variable representing any record of admission to a hospital in Denmark due to self-harm within the past 12 months leading up to the first registered admission to drug use disorder treatment. Using the Central Criminal Register, a dummy variable was created to indicate whether a person had been charged with a crime within the past 12 months, leading up to the first registered treatment admission. The sociodemographic variables include gender, age, civil status (living without a partner or not), not being in education, employment or training, and immigrant status (born in Denmark or not).

### Analyses

Descriptive statistics are reported as percentages for dichotomous variables and means with standard deviations for all other variables. Comparison between groups was done using Nelson-Aalen curves of the cumulative hazard and estimated cumulative incidence functions. Time-to-event analysis for completed suicide was conducted using Fine and Gray’s competing risks analysis [30], in which the cumulative incidence function (CIF); i.e., C_e_ (t) gives the proportion of patients at time *t* who have experienced event *e*, while accounting for the fact that patients can experience another event that prevents event *e* from happening, labeled the competing event or competing risk (e.g., death not caused to suicide will rule out later death caused by suicide).

In the analyses, subjects were considered to have experienced the event if they died due to suicide, to have experienced the competing event, if they died from any other cause, and to be censored if no event took place by December 31st, 2010.

All *p*-values were 2-tailed, and level of significance was assessed as a Type I error with rate of alpha 0.05. All statistical analyses were performed using Stata 15 [31].

In order to compare the rate of suicide among people treated for DUD with the rate in the total population, we drew matched controls from a representative sample of the national population without a history of public funded treatment for DUD or alcohol use disorders. The sample size of the control group is five randomly drawn individuals from the total population (*n* = 139,710), proportionate to each individual enrolled at a DUD treatment center within the time period of this study, using similar gender and age group at enrolment date at the treatment centers. The age groups were 18–27, 28–37, 38–47, and 48–75 years at the time of admission to treatment.

A random enrolment date was generated for the control group. The only restriction made in the matching was that the individuals in the control group did not die before the enrolment date, the year that the follow-up started, and did not have a record of treatment for a drug or alcohol use disorder in the databases. In the control group, the time-to-event outcome variable was created after generating a random enrolment date with a beta distribution.

We analyzed the standardized mortality ratios (SMRs) to estimate the suicide gap between individuals with DUD, recent psychiatric history, or both, compared with a general population sample with neither. Comparisons were made using three categories: individuals in the control group who had past year psychiatric care history, patients treated for DUD with past year psychiatric care, and patients treated for DUD without past year psychiatric care. We used individuals in the control group without past year psychiatric care as the reference group.

## Results

### Descriptive statistics

**Fig. 1:**
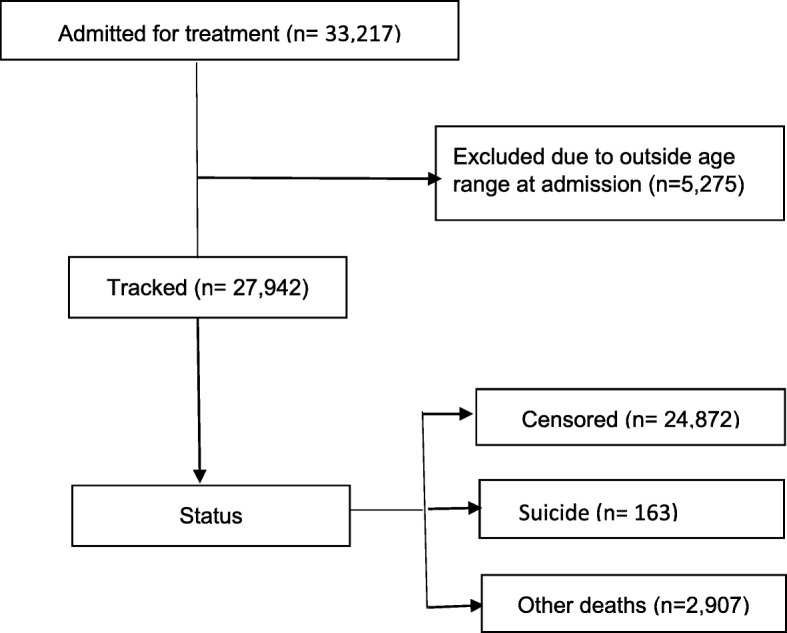
Flow of participants through the study

A cohort of 27,942 patients enrolled in treatment for DUD between 2000 and 2010 were identified in the study sample (see Fig. 1). Table 1 shows baseline demographic and clinical characteristics of individuals in the study. The most commonly registered drugs were cannabis 14,651 (52.4%), opioids 11,131 (39.8%), and central stimulants 8661 (31.0%). The majority of patients were men 21,171 (75.8%), and the mean age was 33.5 (standard deviation [SD] = 10.5). Most of the patients were not in education, employment or training 19,276 (69.0%), were living without a partner 20,204 (72.3%), and had not been treated for DUD previously 15,212 (54.4%).

**Table 1:** Sample Characteristics and Results of Risk Factors Associated With Completed Suicide Among People Treated for Drug Use Disorders Between 2000 and 2010 (*n* = 27,942)

	*n*/percentage (mean/standard deviation)	Sub hazard ratio (95% confidence interval)
Year before drug use disorder treatment variables^1^		
Criminal charges within the past year	11,663 (41.7)	0.86 (0.62, 1.20)
Self-harm within the past year	631 (2.3)	1.48 (0.70, 3.10)
Psychiatric care within the past year	5298 (19.0)	1.96 (1.39, 2.77)
Clinical variables^2^		
Use of opioids	11,131 (39.8)	1.81 (1.23, 2.68)
Use of central stimulants	8661 (31.0)	0.85 (0.58, 1.25)
Use of cannabis	14,651 (52.4)	0.69 (0.50, 0.96)
Use of Methyl​enedioxy​methamphetamine	2023 (7.2)	0.71 (0.34, 1.46)
Alcohol use (problem drinking)	7182 (25.7)	1.56 (1.09, 2.23)
Use of benzodiazepines	4458 (16.0)	1.18 (0.79, 1.76)
Intravenous drug use	15,299 (54.8)	1.23 (0.86, 1.78)
Treatment status^2^		
Previously treated	9623 (34.5)	Reference
Never treated	15,212 (54.4)	1.14 (0.78, 1.67)
Missing information	3107 (11.1)	1.44 (0.84, 2.45)
Socio-demographics^1^		
Female gender	6771 (24.2)	0.71 (0.47, 1.05)
Age (mean/standard deviation)	27,942 (33.5/10.5)	0.97 (0.95, 0.98)
Not in education, employment or training	19,276 (69.0)	1.13 (0.76, 1.70)
Living without partner	20,204 (72.3)	1.10 (0.75, 1.63)
Immigrant^a^	2286 (8.2)	0.78 (0.41, 1.47)

Note: ^a^ not born in Denmark

^1^Based on record linkage

^2^Based on the Registry of Drug Abusers in Treatment

In the sample of people treated for DUD, 163 (0.6%) patients had completed suicide, of which 52.1% had used violent methods, and 47.9% had died from poisonings. In addition, 2907 (10.4%) died due to other causes. Among the controls across both people with and without recent psychiatric history, 111 (0.1%) had completed suicide, of which 85.6% had used violent methods and 14.4% had died from poisonings. Further, 1607 (1.2%) died from other causes.

In the case sample, 50 (31.0%) of suicide completers had received psychiatric care in the past year compared to 15 (13.5%) in the control group. The prevalence of psychiatric care among those who did not commit suicide were 1461 (1.1%) in the control group versus 4619 (18.6%) in patients treated for DUD.

**Fig. 2:**
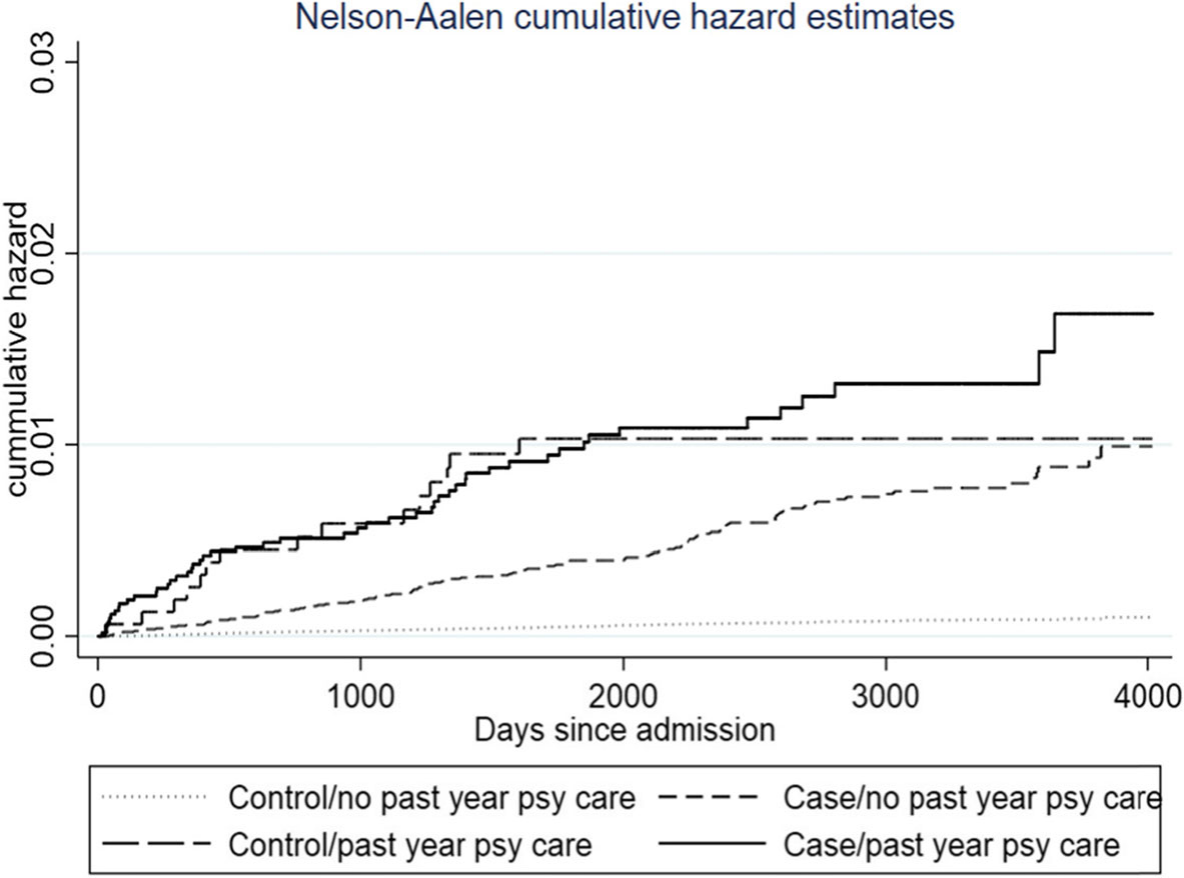
Nelson-Aalen curves for completed suicide between control and case groups with or without past year psychiatric care

Figure 2 shows the Nelson-Aalen cumulative incidence of suicide for all four groups (i.e., [1] people with a history of treatment for DUD without psychiatric history, [2] people with a history of treatment for DUD with psychiatric history, [3] people with no history of treatment for alcohol or DUD without psychiatric history, and [4] people with no history of treatment for alcohol or DUD with psychiatric history.

The Nelson-Aalen estimator plot shows that the risk of dying by suicide differs between the case and control groups, as well as across past year psychiatric care status, where cases with past year psychiatric care had the highest risk of dying from suicide and the controls with no past psychiatric care had the lowest risk.

### Competing-risks regression analysis for risk of suicide among people treated for DUD

**Fig. 3:**
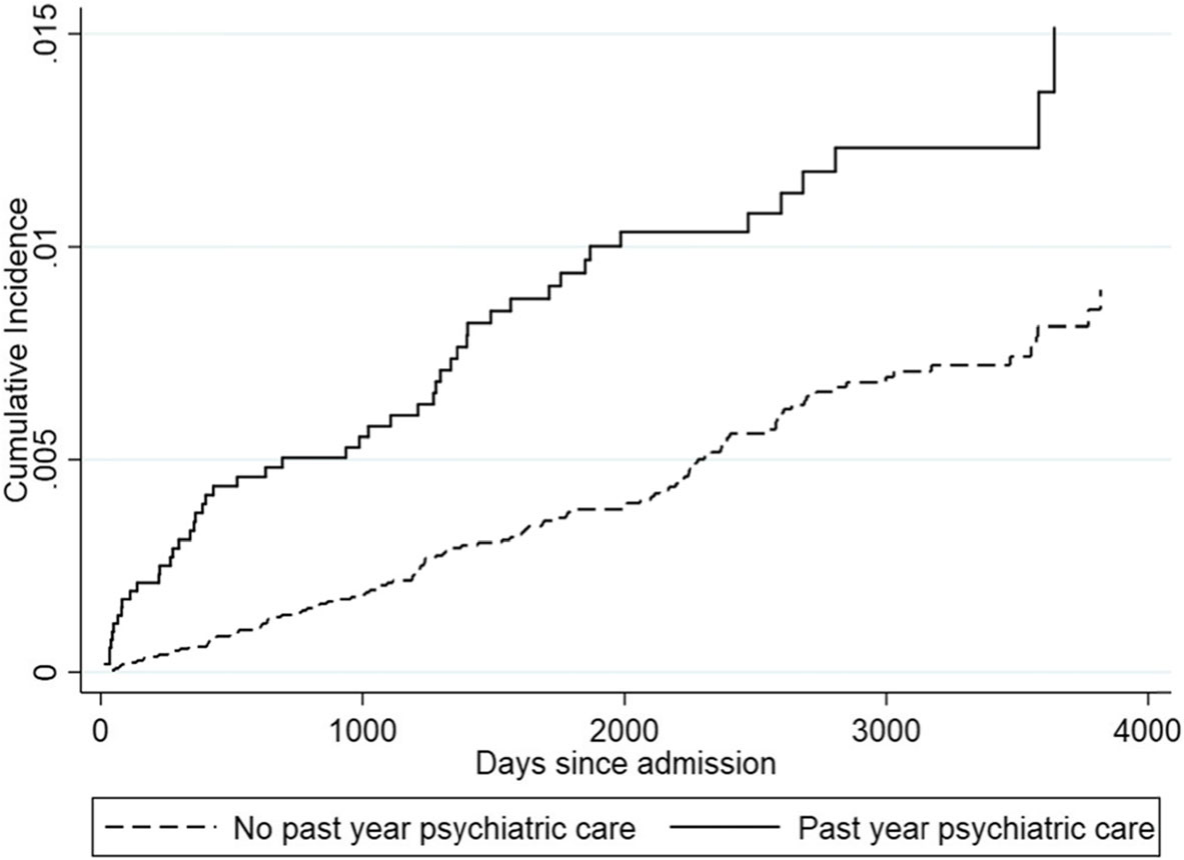
Comparative cumulative incidence of completed suicide with and without past year psychiatric care for individuals with DUD

The mean time at risk for completed suicide was 5.8 years. The results from the competing-risks regression model (Table 1) shows that the risk of completed suicide was higher among those who were in psychiatric care in the year prior to treatment for DUD (HR = 1.96, 95% CI: 1.39, 2.77). The two substance-related risk factors associated with higher risk of completed suicide were opioid use (HR = 1.81, 95% CI: 1.23, 2.68) and alcohol (HR = 1.56, 95% CI: 1.09, 2.23), whereas use of cannabis was associated with lower risk of completed suicide (HR = 0.69, 95% CI: 0.50, 0.96). Among the socio-demographic variables, older age (HR = 0.97, 95% CI: 0.95, 0.98) was associated with lower risk of suicide. Figure 3 shows the adjusted cumulative incidence function by psychiatric care history based on the competing risks regression.

### Comparison to general population sample with and without psychiatric history

Comparisons between groups are shown in Table 2.

**Table 2:** Standardized Mortality ratios due to suicide (SMR) of Cases and Controls With and Without Past Year Psychiatric Care

	Control without past psychiatric care (*n* = 138,136)	Control with past psychiatric care (*n* = 1574)	Case with past psychiatric care (*n* = 5298)	Case without past psychiatric care (*n* = 22,644)
	SMR(95%CI)	SMR(95%CI)	SMR(95%CI)	SMR(95%CI)
Men	(ref)	12.40 (5.08–19.74)	12.70 (8.60–16.79)	6.63 (5.28–7.98)
Women	(ref)	42.92 (0.86–84.98)	39.27 (17.92–60.63)	19.55 (10.98–28.11)
All	(ref)	13.61 (6.72–20.50)	13.48 (9.75–17.22)	7.13 (5.81–8.44)

Cases: Patients Treated for Drug Use Disorder

In the following comparisons, we shall refer to age- and gender-matched controls without psychiatric history as the “reference group”. Patients treated for DUD without past year psychiatric care history were more likely to commit suicide than the reference group (SMR = 7.13, 95% CI: 5.81, 8.44). Thus, the risk for suicide among people with a history of DUD and no psychiatric history was 7.13 times higher compared with a person in the general population with no recent history of psychiatric care. The suicide SMR for individuals with DUD and recent psychiatric history was 13.48 (95% CI: 9.75, 17.22).

Finally, individuals taken from the general population with a history of psychiatric care in the last 365 days before their random enrolment date were more likely to commit suicide (SMR = 13.61, 95% CI: 6.72, 20.50) than the reference group.

## Discussion

### Summary of findings

Using multiple Danish national registers, we examined whether excess mortality due to suicide exists among people treated for DUD, and identified risk factors associated with completed suicide between 2000 and 2010. We also compared the prevalence of suicide between individuals treated for DUD and the general population.

Patients who had been in treatment for DUD, but who did not seek psychiatric care in the past year had more than a sevenfold increase in risk of suicide after admission to treatment, in comparison to age- and gender matched individuals from the general population without a history of recent psychiatric care. This is a substantial increase in risk, which is in line with findings from psychological autopsies [5], and the 95% confidence interval between five and eight clearly indicates a statistically significant difference.

Patients who had been in treatment for a DUD with past psychiatric care had a more than 13 times higher risk of committing suicide compared with gender or age-matched individuals with no history of treatment for substance use disorders and psychiatric care in the general population. However, the high relative elevation must be considered in light of the low base-rate of completed suicide, and it must be remembered that among the individuals that we tracked for up to ten years, less than 1% took their own lives (compare also [32]).

In addition, it should be noted that people from the general population with a recent history of psychiatric care had practically the same elevation in risk as patients with both a history of treatment for DUD and recent psychiatric care (SMR = 13.6).

Our findings highlight some key risk factors for suicide among people seeking treatment for DUD. We found that younger age, past year history of psychiatric care, use of opioids, and use of alcohol were all associated with increased risk of suicide. Use of cannabis was associated with a lower risk of suicide. Past psychiatric care was associated with a higher risk of suicide in our cohort. As would be expected, mental health problems were associated with an elevated suicide risk in both the DUD cohort and among the controls, a finding also reported by Cavanagh et al. [33].

Our finding that opioid use was a strong predictor of completed suicide is consistent with other studies [7, 34, 35], even if those other studies have assessed suicidal ideation and attempts, rather than completed suicide. In our context, opioid use was part of a drug problem that had led to treatment, meaning that our findings may not be relevant for opioid use among pain patients (compare [36]).

Our finding that alcohol use was a strong predictor of completed suicide is also consistent with other studies [37, 38]. For instance, one in five people who committed suicide in an Australian psychological autopsy study were found to have an alcohol use disorder [39]. In addition, alcohol intoxication is associated with methods of increased lethality when attempting suicide, i.e. methods that have a higher risk of a fatal outcome [40]. However, this study adds to the literature by showing that even among people with other DUD, alcohol is an independent contributor to suicide risk.

Our finding that cannabis was associated with lower risk of completed suicide was unexpected [41, 42]. It is possible that third variable confounding underlie these negative correlations. However, some research suggests at least one active component in cannabis, namely cannabidiol [43], can have beneficial effects on substance use disorders, by reducing drug seeking behaviour and symptoms of anxiety [43]. This may in turn reduce the risk of completed suicide in the context of multiple types of DUD. It is also possible that the general loss of initiative associated with cannabis use may indirectly influence suicidal behaviour as well [44].

Our findings of no association between self-harm and completed suicide is contrary to other studies (see 12). It is possible that we did not capture self-harm with sufficient precision, or that our patient group did not present with self-harm at hospital-based clinics, including emergency departments, but rather with symptoms of intoxication or withdrawal.

### Implications for practice

There is growing evidence that patients with DUD who experience mental health problems may be helped by interventions that are integrated with substance abuse treatment. At least one meta-analysis [45], as well as more recent clinical trials [46–48], indicate that both mental health problems and substance use disorders are receptive to psychotherapy as a mode of treatment. Evidence from large-scale trials indicate that integrated services for people with co-morbid substance use disorders and mental health problems can be implemented in routine clinical settings [49].

Furthermore, there is evidence that antidepressants can be helpful for people with co-morbid depression and substance use disorders, even if the effects are larger when patients are abstinent before being treated [50, 51] and the quality of the evidence is mixed [52]. In this context, benefits and risks should be carefully weighted: There is a risk that antidepressants may become part of a lethal cocktail of substances leading to a fatal overdose [53]. However, this relationship is complicated, as depression and anxiety disorders may themselves be associated with overdose risk, and this risk may be attenuated by treatment with antidepressants that is continuous over a longer period of time [54].

Finally, other studies show that mental health problems can be validly assessed among people undergoing treatment for DUD with use of self-report instruments [55–57]. As such, identification of co-existing psychopathology should be highlighted even more as a potential first step towards suicide prevention.

### Strengths and limitations

Some limitations must be noted for this study. First, as with any register-based study, we were not able to provide direct quality control over the process of data collection. Secondly, and perhaps more importantly, suicides by poisonings may be difficult to discriminate from overdoses [58]. This could especially lead to an under-estimation of the association between opioid use and suicide, as opioids are the drugs that are primarily involved in accidental poisonings [58].

In the present study, the case definition of suicide only included ICD-10 causes of death codes for intentional self-inflicted poisoning or injury (X60-X84), and sequelae of self-harm (Y87.0). A systematic review from 2012 concluded that suicide deaths are generally under-reported [59]. In this review, the level of under-reporting varied between different primary studies, but high quality studies tended to report less under-reporting than studies of poorer quality. As such, it is likely that our case definition leads to lower bound estimates of suicide deaths and may dilute estimated associations. A potential solution to under-reporting is redistribution of ICD-10 death codes which may contain suicide deaths (such as undetermined intent injury codes (Y10-Y34), and exposure to undetermined factors (X59) [2]). However, a recent Norwegian national registry study covering death certificates for the years 2005–2014 reported that redistribution of X59 codes which constituted 26% of all injury deaths, only changed the suicide estimates by 2 percentage points (60). In the same study, 12% of all injury deaths were assigned undetermined intent injury codes (Y10-Y34). The Norwegian and Danish registries of Causes of Death share many similarities, use the same coding system (ICD-10), and are both of high quality. It is therefore unlikely that redistribution of death codes would change our overall findings to a great extent, perhaps with exception of actual intentional over-doses being assigned as undetermined intent (Y10-Y15). Finally, while it was a strength to include a matched control group in this study, because it allowed us to compare the prevalence of suicide between individuals who attended DUD treatment and individuals who did not, we cannot exclude the possibility of selection bias due to the chosen matching strategy.

## Conclusions

Risk of suicide is increased among people with drug use disorders compared with the general population, although the number of suicides among people with drug use disorder is relatively small compared with drug-related deaths. The main risk factors for suicide in patients with drug use disorders are a history of mental health problems, and opioid and alcohol use.

## Data Availability

The datasets analyzed for the present study are the property of the Danish government, and are stored on servers owned by Statistics Denmark. The Danish Board of Health data (https://sundhedsdatastyrelsen.dk/da), and Statistics Denmark (https://www.dst.dk/da/) can provide information about how to apply for access.

## Abbreviations

CI: Confidence interval
DUD: Drug use disorder
HR: Hazard ratio
ICD-10: International classification of Diseases, Tenth Revision
SMR: Standardized mortality ratios due to suicide
